# Chemical Modifications of an Insect Immune Resolvin, EpOME, to a Broad-Spectrum Lepidopteran-Specific Insecticide

**DOI:** 10.3390/insects17060588

**Published:** 2026-06-04

**Authors:** Falguni Khan, Taegeun Song, Anders Vik, Niayesh Shahmohammadi, Yonggyun Kim

**Affiliations:** 1 School of Smart-Green, College of Life Sciences & Engineering, Gyeongkuk National University, Andong 36279, Republic of Korea; falgunikhan2942@gmail.com (F.K.); xorms5095@naver.com (T.S.); niayeshshahmohammadi@gmail.com (N.S.); 2 Department of Pharmacy, Section for Pharmaceutical Chemistry, University of Oslo, 0371 Oslo, Norway; anders.vik@farmasi.uio.no

**Keywords:** EpOME, alkoxide analog, stereoisomer, immunity, insecticide

## Abstract

EpOMEs suppress excessive and unnecessary immune responses at late infection stage in insects. This immune resolvin activity of EpOMEs includes the cell lysis of hemocytes by causing apoptosis. This cytolytic toxicity of EpOMEs suggests a potential insecticidal activity of their stable analogs. In fact, a propoxy derivative of 12,13-EpOME called AS56 mimicked EpOME activity by suppressing immune responses and exhibited insecticidal activity against a lepidopteran insect, *Spodoptera exigua*. This study evaluated the potency of the EpOME analogs by modifying their functional groups. PD28 is a racemic mixture of propoxy analog at the twelfth carbon. FD25 is a racemic butoxy mixture at the twelfth carbon of AS56. AS61 is saturated at the ninth carbon of AS56. The effects of the EpOME analogs were assessed on two cellular immune responses: hemocyte-spreading and nodule formation. All four analogs inhibited the cellular immune responses; however, AS56 was the most potent in inhibiting the immune responses. FD25 and AS61 were significantly less potent compared to AS56, suggesting crucial roles of the double bond at the ninth carbon and the propoxy chain at the twelfth carbon. The stronger inhibitory activity of AS56 compared to PD28 also suggests the functional role of the stereoisomeric form in physiological functions. The cytotoxicity of AS56 was also the most potent among the analogs, suggesting its insecticidal activity. Subsequent insecticidal bioassays on AS56 toxicity were performed against different insect species. These EpOME analogs were potent against lepidopteran insects (*S. exigua* and *Plutella xylostella*) but not against coleopteran (*Tenebrio molitor*) or thysanopteran (*Frankliniella occidentalis*) species. Among the EpOME analogs, AS56 was the most toxic against the lepidopteran insects. Spraying AS56 in cabbage fields infested by lepidopteran insects led to acute and high control efficacy against two lepidopteran pests, similar to that of a chemical insecticide, fluxametamide. The AS56-intoxicated larvae exhibited over-excitation in their behavior around 12 h post-treatment. This study indicates that AS56 exhibits an oral toxicity against lepidopteran insects with its cytotoxicity and behavioral over-excitation.

## Simple Summary

EpOME is an oxylipin that mediates insect immunity and plays a crucial role in turning off the unnecessary and excessive immune responses at late infection stage. Its cytotoxicity was exploited to develop a potent insecticidal compound. A novel compound, called AS56, has been devised by altering EpOME by methylating the terminal carboxyl group and replacing epoxide with alkoxide. This study supports the compound structures in double bond position and alkoxide stereoisomer in performing the physiological functions. Surprisingly, this compound exhibits high and specific insecticidal activity against lepidopteran insect pests. Its application to cabbage crops in the field conditions showed high control efficacies against two pests (*Spodoptera exigua* and *Plutella xylostella*), similar to a commercial insecticide.

## 1. Introduction

Oxylipins are a diverse family of bioactive lipid signaling molecules derived from the oxygenation of polyunsaturated fatty acids, acting as crucial regulators in nearly all organisms from plants to humans [1]. Two kinds of oxylipins play crucial roles in mediating reproduction and immunity in insects: eicosanoids and epoxyoctadecamonoenoic acids (EpOMEs) [2]. Eicosanoids are usually derived from arachidonic acid (AA), which is released from phospholipids by the catalytic activity of phospholipase A_2_ (PLA_2_) [3]. AA is then oxygenated into three different eicosanoid groups as prostaglandins (PGs), leukotrienes, and epoxyeicosatrienoic acids, which are detected and regulated in terms of their levels depending on physiological conditions [4,5,6]. EpOMEs, including coronaric acid (9,10-EpOME) and vernolic acid (12,13-EpOME), are derived from linoleic acid (LA) by the catalytic activities of specific cytochrome P450 monooxygenases [7]. EpOMEs are degraded into 9,10-dihydroxy-octadecamonoenoate (9,10-DiHOME) and 12,13-dihydroxy-octadecamonoenoate (12,13-DiHOME) by soluble epoxide hydrolase (sEH). In insects, EpOME and DiHOME titers fluctuate in response to pathogen infections in species, such as two moths (*Spodoptera exigua* and *Maruca vitrata*) and a thrips (*Frankliniella occidentalis*) [8,9,10].

All three types of eicosanoids mediate immune responses in insects by acting on hemocytes and the fat body, in which the molecular action of prostaglandin E_2_ (PGE_2_) is well understood in its molecular action through the identification of its specific receptor [4,11]. Insects defend against microbial pathogens through innate immune responses following the specific recognition of pathogen-associated molecular patterns [12]. The recognition signal is then propagated to nearby immune effectors using oxylipins and other immune mediators [13]. Hemocytes and fat body are the main immune-associated tissues and perform cellular and humoral immune responses against the pathogens [14]. In a lepidopteran insect, *S. exigua*, the PGE_2_ receptor was localized in specific hemocyte types via single-cell RNA-Seq analysis and is associated with a specific trimeric G protein containing Gαs, leading to the upregulation of cAMP levels [15,16]. PGE_2_ influences different physiological processes in insects. For example, a mutant of the PGE_2_ receptor dies as a pharate adult with insufficient tracheal development in *Drosophila melanogaster*, which can be rescued by supplying high oxygen [17]. In reproduction, PGE_2_ mediates oogenesis by stimulating nurse cell dumping during vitellogenesis [18] and by inducing chorionic genes in mosquitoes and thrips [19,20].

Like in mammals, EpOMEs and DiHOMEs have been speculated to play a crucial role in the immune response of insects [21]. Indeed, they were detected at all the developmental stages of the mosquito, *Culex quinquefasciatus*. Feeding an sEH inhibitor increased EpOME levels in the mosquito midgut and reduced the total number of bacteria in the lumen, suggesting that EpOMEs are associated with insect immunity [22]. The role of EpOMEs in insect immune responses was further analyzed in the immune-challenged larvae of *S. exigua*, which possess specific EpOME synthase and sEH [5]. These two regio-isomers of EpOMEs suppress the cellular and humoral immune responses. Their immunosuppressive effects were further supported by treatment with urea-based sEH inhibitors, which inhibited the cellular and humoral immune responses of *S. exigua* by increasing EpOME levels in response to immune challenges. Thus, EpOMEs facilitate anti-inflammatory responses in insects. Interestingly, EpOMEs are synthesized at a relatively late infection stage, while PGE_2_ and other eicosanoids are acutely synthesized and induce immune responses [9]. This suggests that EpOMEs act as immune resolvins in insects to prevent unnecessary and excessive self-targeting responses.

The immunosuppressive activity of EpOMEs has been used to enhance the virulence of entomopathogens, such as *Bacillus thuringiensis* and Autographa californica multiple nucleopolyhedrosis virus [5,23]. On the other hand, the cytotoxic activity of EpOMEs against insect cells, like hemocytes and Sf9 cells, has suggested direct insecticidal activity. Indeed, EpOME analogs (including AS61 with a saturated 9–10 double bond, FD25 with a butoxy chain, and PD28 as a racemic mixture) were synthesized by replacing the epoxide group with an alkoxide at the twelfth carbon position, among which the 12*R*-propoxyl regio-isomer called AS56 was the most potent in killing the lepidopteran insects [24]. However, the significance of the double bond at ninth carbon and the alkoxide chain length at the twelfth carbon were not fully understood in AS56 with respect to insecticidal activity. The toxic activity of AS56 needed to be analyzed in other biological contexts. Finally, this study analyzed the field efficacy of AS56 against insect pests via spray application, compared to a commercial insecticide reference.

## 2. Materials and Methods

### 2.1. Insect Rearing

Larvae of *S. exigua* were originally collected from Welsh onion fields in Andong, Republic of Korea, and subsequently reared under controlled laboratory conditions (25 ± 2 °C, 70 ± 5% relative humidity, and a 16:8 h light:dark photoperiod). Larvae were reared on cabbage and underwent five instars (L1–L5). Larvae of *Plutella xylostella* were also reared on cabbage leaves under the same environmental conditions and underwent four instars (L1–L4). Adults were provided with a 10% sucrose solution for oviposition.

A laboratory colony of *F. occidentalis* was established with individuals collected from a hot pepper field in Andong, Republic of Korea, and maintained under the same laboratory conditions. Larvae and adults were fed germinated kidney beans (*Phaseolus coccineus* L., cv. Kangnam) purchased from the Yechon Agricultural Cooperation, Yechon, Republic of Korea. Newly hatched larvae were transferred daily to breeding dishes (100 × 40 mm; SPL Life Sciences, Pocheon, Republic of Korea). *T. molitor* larvae were obtained from Bio Utility, Inc. and reared on wheat bran according to the method described by Liu et al. [25]. Rearing conditions were maintained at 25 ± 2 °C, 60 ± 5% relative humidity, and a 16:8 h light:dark photoperiod. Under these conditions, *T. molitor* larvae underwent 12 larval instars (L1–L12).

### 2.2. Chemicals

PGE_2_ ((5*Z*,11*α*,13*E*,15*S*)-11,15-dihydroxy-9-oxoprosta-5,13-dienoic acid) and benzylideneacetone were purchased from Sigma-Aldrich Korea (Seoul, Republic of Korea) and dissolved in 100% dimethyl sulfoxide (DMSO). Fura-8AM was purchased from AAT Bioquest (Sunnyvale, CA, USA) and dissolved in 100% DMSO. Phosphate-buffered saline (PBS) was prepared with 100 mM phosphoric acid and adjusted to pH 7.4. Fluorescein isothiocyanate (FITC)-tagged phalloidin (Alexa Fluor 488 phalloidin) and 4′,6-diamidino-2-phenylindole (DAPI) were purchased from Thermo Fisher Scientific (Seoul, Republic of Korea). Anticoagulant buffer (ACB) consisting of 186 mM NaCl, 17 mM Na_2_EDTA, and 41 mM citric acid was prepared, and its pH was adjusted to 4.5 with HCl. MTT (3-[4,5-dimethylthiazole-2-yl]-2,5-diphenyl tetrazolium bromide) was purchased from Sigma-Aldrich (Seoul, Republic of Korea).

### 2.3. Hemocyte-Spreading Behavior Using an Immunofluorescence Assay (IFA)

Hemolymph (approximately 150 μL) was collected from five L5 *S. exigua* larvae via proleg incision and immediately mixed with 350 μL of ACB. The mixture was placed on ice for 20 min to stabilize hemocytes and minimize spontaneous spreading. After centrifugation at 1000× *g* for 2 min at 4 °C, 250 μL of the supernatant was removed, and the remaining hemocyte suspension was gently mixed with 250 μL of TC100 insect tissue culture medium (Welgene, Gyeongsan, Republic of Korea). Subsequently, 10 μL of the hemocyte suspension was placed on a glass coverslip in a wet chamber under dark conditions. To assess the effects of EpOME analogs, the hemocyte suspension was treated with each compound (AS56, AS61, FD25, or PD28) at 0, 1, 10, or 100 µg/mL along with PGE_2_ (10 µg/mL), followed by incubation for 1 h at room temperature (RT). Afterward, the cells were washed three times with PBS. The cells were fixed with 4% paraformaldehyde for 10 min at RT, followed by three times washing with PBS. Then, the cells were permeabilized with 0.2% Triton X-100 in PBS for 15 min at RT. After washing three times in PBS, the cells were blocked with 10% BSA in PBS for 10 min at RT. Following washing with PBS three times, the cells were exposed to 1% Alexa Fluor 488 phalloidin in PBS for 1 h at RT. After three washes with PBS, the cells were treated with 0.1% DAPI in PBS for 5 min. After three times washing PBS, the cells were examined under a fluorescence microscope (DM2500; Leica, Wetzlar, Germany) at 400× magnification. Each experimental treatment was replicated three times for validation. For each replicate, all visible hemocytes in the treatment field were counted. The FITC fluorescence signal intensity was divided by the DAPI signal intensity to normalize the difference in the cell numbers. Each treatment was replicated with three different hemocyte preparations from different insect samples.

### 2.4. Nodulation Assay

L5 larvae of *S. exigua* were used for nodule formation in response to the hemocoelic injection of *Escherichia coli* (5 × 10^4^ cell/larvae). After 12 h of incubation at RT, larvae were dissected along the dorsal line, and melanized nodules formed in the hemocoel were observed and counted under a stereoscopic microscope (Stemi SV11, Zeiss, Jena, Germany) at 50× magnification. The number of nodules per larva was recorded. To test the inhibitory activity of EpOME analogs on cellular immunity, each larva was injected with 1 µL of the test compound solution (1 µg/larva) in combination with PGE_2_ (1 µg/larva) through the proleg using a microsyringe. Control larvae were injected with an equal volume of DMSO. Each treatment was independently replicated three times, with each replicate consisting of three larvae per treatment.

### 2.5. Ca^2+^ Signaling Assay in Hemocytes

Hemocytes were prepared as described in IFA. Subsequently, 10 μL of the hemocyte suspension was placed onto a glass coverslip in a moist chamber. The cell suspension was then treated with each EpOME analog (AS56, AS61, FD25, or PD28) at 100 µg/mL along with PGE_2_ (10 µg/mL) and incubated at RT for 1 h. After incubation, 2 μL of Fura-8 AM (1 mM) was added to each sample and incubated for 2 h at RT in the dark. The cells were fixed with 4% paraformaldehyde for 10 min at RT and washed three times with PBS. Permeabilization was carried out using 0.2% Triton X-100 in PBS for 10 min, followed by additional PBS washes. Fura-8 AM-positive cells, indicating intracellular Ca^2+^ levels, were observed under the fluorescence microscope at 200× magnification. Fluorescence intensity changes were analyzed using ImageJ software v1.54g on 24 February 2026 (https://imagej.net/ij/). Each treatment was independently replicated three times. For each replicate, all visible hemocytes in the treatment field were counted. The numbers of three independent replicates were averaged for analysis.

### 2.6. Total Hemocyte Count (THC) and Differential Hemocyte Count (DHC)

L5 larvae of *S. exigua* were injected with the test compound at a dose of 1 μg per larva. Hemolymph was collected 4 h post-injection by puncturing the proleg with a sterile capillary tube. Approximately 50 μL of hemolymph was obtained from each larva and immediately diluted with anticoagulant buffer to prevent coagulation. The diluted samples were loaded onto a hemocytometer, and THC was performed using a phase-contrast microscope (CKX31, Olympus, Tokyo, Japan) at 400× magnification. The hemocytes were also classified into four different morphological types based on the criteria indicated by Khan et al. [16]: granulocyte (GR), plasmatocyte (PL), oenocytoid (OE), and spherulocyte (SP). DHC was estimated by their relative proportions in percentages. A total of three larvae were analyzed in each replication, and the experiment was replicated three times for each treatment.

### 2.7. Classification of Hemocytes Using Fluorescence In Situ Hybridization (FISH) Marker

FISH was performed on hemocytes collected from L5 *S. exigua* larvae. Hemocytes were prepared as described in IFA. An aliquot (20 µL) of the hemocyte suspension was placed onto a glass slide in a dark, humid chamber. Hemocytes were treated with AS56 (100 µg/mL) in combination with PGE_2_ (10 µg/mL) and incubated for 1 h at RT. Cells were then fixed with 4% paraformaldehyde for 10 min at RT, washed three times with PBS, permeabilized with 0.2% Triton X-100 in PBS for 10 min, and then followed by three washes with PBS. Samples were rinsed in 2× saline–sodium citrate (SSC) buffer and incubated at 42 °C with 25 μL of pre-hybridization buffer (2 μL yeast tRNA, 2 μL 20× SSC, 4 μL dextran sulfate, 2.5 μL 10% SDS, and 14.5 μL deionized water) in a dark, humid chamber for 1 h. After pre-hybridization, the buffer was replaced with hybridization buffer (5 µL deionized formamide and 1 µL fluorescein-labeled oligonucleotide probe in 19 µL pre-hybridization buffer). DNA oligonucleotide probes were labeled at the 5′ end with fluorescein amidite (FAM) and purified by high-performance liquid chromatography (Bioneer, Daejeon, Republic of Korea). To identify hemocyte types, antisense probes complementary to target mRNAs were used, with corresponding sense probes serving as negative controls (Table S1). Slides were covered with RNase-free coverslips and incubated overnight (16 h) in a humid chamber at 42 °C. After hybridization, hemocytes were washed twice with 4× SSC for 10 min each, incubated in 4× SSC containing 1% Triton X-100 for 5 min at RT, washed twice again with 4× SSC for 10 min each, and finally washed once with 2× SSC. Samples were air-dried, mounted in PBS/glycerol (1:1, *v*/*v*), and covered with a coverslip. Observations were performed under a fluorescence microscope at 200× magnification.

### 2.8. Cytotoxicity Assay of EpOME Analogs Using MTT Test

The cytotoxicity of EpOME analogs was evaluated in Sf9 cells using an MTT assay. Sf9 cells were seeded into 96-well plates at a density of 3.3 × 10^4^ cells per well and incubated at 28 °C for 24 h. Cells were then treated with the test EpOME analogs, while untreated cells served as negative controls, benzylideneacetone (1 µM) as the positive control, and cells treated with DMSO served as the solvent control. After 24 h incubation at 28 °C, 10 μL of MTT solution (5 mg/mL in PBS) was added to each well, followed by an additional 8 h of incubation under the same conditions. The resulting formazan crystals were dissolved in 50 μL of DMSO, and absorbance was measured at 570 nm using a microplate reader (Victor Multi-label Plate Reader, PerkinElmer, Shelton, CT, USA). Each treatment was performed in triplicate wells and repeated in three independent biological experiments.

### 2.9. Cytotoxicity Test of EpOME Analogs Using TUNEL Assay

Apoptotic cells were detected using an in situ Cell Death Detection kit (Abcam, Cambridge, UK) following the manufacturer’s instructions. Hemocytes were prepared as described in IFA. Then, 10 μL of the hemocyte suspension was placed onto a glass coverslip in a wet chamber under dark conditions. Collected hemocytes were then incubated in a reaction mixture containing 10 µM 5-bromouridine (BrdU) and terminal deoxynucleotidyl transferase for 1.5 h at RT to label DNA fragmentation sites. To assess the effects of EpOME analogs, the hemocyte suspension was treated with each compound (AS56, AS61, FD25, or PD28) at 100 µg/mL along with PGE_2_ (10 µg/mL), followed by incubation for 1 h at RT. The cells were fixed with 4% paraformaldehyde for 10 min at RT and washed three times with PBS. Permeabilization was carried out with 0.2% Triton X-100 in PBS for 10 min. After washing, cells were blocked with 10% bovine serum albumin in PBS for 10 min at RT. Hemocytes were then stained with 1% Alexa Fluor 488 phalloidin in PBS for 1 h at RT to visualize F-actin, followed by three PBS washes. Nuclei were counterstained with 0.1% DAPI in PBS for 5 min. Following three final washes with PBS, samples were mounted on glass slides in a glycerol:PBS solution and visualized under a fluorescence microscope (DFC450C, Leica, Germany) using FITC and DAPI filter sets. Each treatment was independently replicated three times. Fluorescence intensity was quantified using ImageJ software.

### 2.10. Apoptosis Determination Using an Antibody Specific to Cleaved Caspase-3

To determine apoptosis, an immunofluorescence assay (IFA) was performed using a specific antibody against cleaved caspase-3 (Asp175) (#9661, Cell Signaling Technology, Boston, MA, USA). Hemolymph was collected into ACB as described above. Subsequently, 10 μL of hemocyte suspension was placed onto a glass slide in a humid chamber and treated with AS56 at 100 µg/mL, followed by incubation for 1 h at RT. After washing the cells with 1X PBS, cells were fixed with 4% formaldehyde for 1 h RT. After washing three times with PBS, the cells were permeabilized with 0.1% Triton X-100 in PBS for 30 min at RT. Following three additional washes with PBS, the samples were blocked with 10% BSA in PBS for 30 min. The hemocytes were then incubated for 2 h at RT in a humid chamber with an antibody against cleaved caspase-3. After careful washing with PBS, the samples were incubated for 2 h at RT with 1% FITC-conjugated anti-rabbit secondary antibody (Thermo Fisher Scientific) diluted in PBS. Following three washes with PBS, the samples were mounted in 50% glycerol, covered with a cover glass, and observed under the fluorescence microscope at 200× magnification. Each treatment was conducted with three biological replicates using different individual insects.

### 2.11. Functional Binding Affinity Measurement of EpOME Analogs to Their Receptor

The equilibrium dissociation constant (K_D_) was calculated using the percentage of Ca^2+^ signal intensity. Because the Ca^2+^ response showed a concentration-dependent decrease in signal intensity (inhibitory-type response), raw fluorescence values were transformed into a binding-style format prior to affinity analysis. The transformation was performed by assuming a direct 1:1 antagonist–receptor relationship using the equation transformed $\;\%\;\mathrm{binding}\;=\frac{\left(Y\max-Y\right)}{\left(Y\max-Y\min\right)}\;\times \;100$, where Y represents the measured FITC fluorescence intensity at a given EpOME concentration; Ymax is the maximum fluorescence signal (typically observed at the lowest EpOME concentration); and Ymin is the minimum fluorescence signal (observed at saturating EpOME concentrations). This transformation converts inhibitory Ca^2+^ signaling data into a monotonically increasing binding curve ranging from 0 to 100%. K_D_ calculation was performed using the transformed % binding values by nonlinear regression in GraphPad Prism v8.0.1 (Boston, MA, USA). Data were fitted to a one-site specific binding model according to the equation $Y\;=\;\frac{\left(B_{\max}\;\times \;X\right)}{\left(K_{D}\;+\;X\right)}$, where Y is the transformed % binding; X is the EpOME concentration; B_max_ is the maximal binding response; and K_D_ is the apparent dissociation constant. Nonlinear curve fitting was performed using least-squares regression, and goodness-of-fit parameters, including R^2^, were evaluated to assess model accuracy. The resulting K_D_ values represent the apparent affinity of EpOME for Ca^2+^ signaling modulation under the experimental conditions.

### 2.12. Insecticidal Bioassay Using a Leaf-Dipping Method and Measurement of Median Lethal Concentration (LC_50_)

All test chemicals were dissolved in DMSO at a concentration of 10,000 ppm. These stock solutions were subsequently diluted with distilled water. For feeding assays, a cabbage piece (1 × 2 cm) was used for *S. exigua*, *P. xylostella*, or *T. castaneum*, while a sprouted bean was used for *F. occidentalis*. Each diet piece was immersed in the test solution for 3 min, then air-dried to remove excess liquid. The treated diet was placed in a 9 cm Petri dish lined with filter paper to prevent excessive moisture. Each assay included 10 individuals per dish with three replicates. LC_50_ values were estimated based on mortality at three days after treatment using Probit analysis of SAS program v9.4 [26].

### 2.13. Determination of Spraying Volume of the AS56 Suspension Using a Pot Assay

Young cabbages with an average height of approximately 10 cm were individually planted in pots (9.3 cm height × 9.7 cm diameter). Each cabbage was infested with 30 young larvae of *S. exigua* or *P. xylostella*. An AS56 solution at 500 ppm was sprayed onto the cabbage at three different volumes. Each treatment was replicated with three pots. Mortality was estimated at three days after treatment.

### 2.14. Control Efficacy Test Using a Field Assay

The field experiments were conducted in a randomized complete block design with different treatments. There were approximately fourteen rows, where the row-to-row distance was approximately 30 cm and the plant-to-plant distance was 25 cm. In each row, ten cabbage plants were planted. Three cabbages were randomly selected in each row for three treatments, in which three replications per treatment were separated into three rows. The test compound at 500 ppm was sprayed at 6 mL per plant. A commercial insecticide, fluxametamide (500 ppm, Captain^®^, Kyungnong, Seoul, Republic of Korea), was also sprayed at the same volume. Surviving larvae were counted at 3 and 7 days after treatment and used for estimating control efficacy compared to the untreated control.

### 2.15. Behavior Monitoring and Automatic Digitization

Individual L5 larvae were injected with 1 µg of AS56 and used for observation of continuous locomotion for 24 h in each trial. The observation system was installed on an optic fiber microscope (ACA1300-60gm NIR, Basler, Inc., Ahrensburg, Germany) consisting of an observation arena, camera, computer, and light. The observation arena was a 9 cm Petri dish with a central food provision (2 cm diameter). The temperature was 26.0 ± 1.5 °C and the humidity was 70.0 ± 5.0% in the observation room. The photophase (14 h) and scotophase (10 h) were provided with white and red LEDs, respectively. To avoid confounding effects between the transfer of test female larvae and light phase changes, the test females were introduced to the observation arena 1 h before the start of the photophase for acclimation. Digital observation was conducted continuously for 24 h immediately after the lights were turned on in the light cycle. Behavioral tracks were recorded with an image resolution of 1280 × 1024 at 30 frames per second with a program (Ethovision XT18, Noldus, Inc., Wageningen, The Netherlands). Walking distance (WD), retention time in the food zone (RTF), and rotation frequency (RF) were recorded as movement parameters and calculated every hour. WD was defined as the total walking distance per hour for the test individual. RTF was the retention time (min) spent in the food-provision area per hour. RF was calculated as the frequency of the head-angle change (without considering direction) per hour.

### 2.16. Statistical Analysis

All studies were subjected to one-way ANOVA using PROC GLM of the SAS program. Means were compared with the least significant difference (LSD) test. This study was conducted with three biologically independent replicates, and the results were plotted as mean ± standard error using SigmaPlot v10.0.

## 3. Results

### 3.1. AS56 as an Insect Immunosuppressant

**Figure 1:**
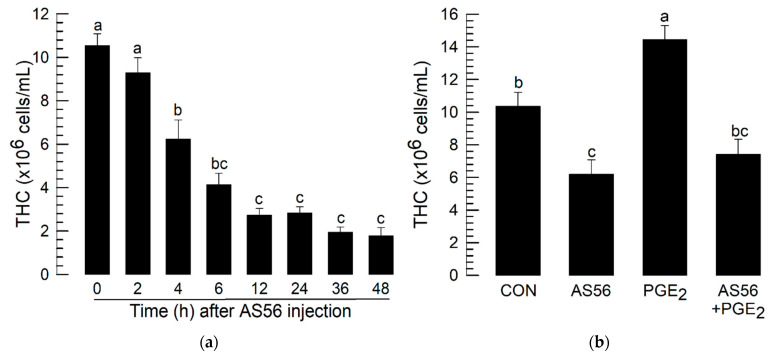

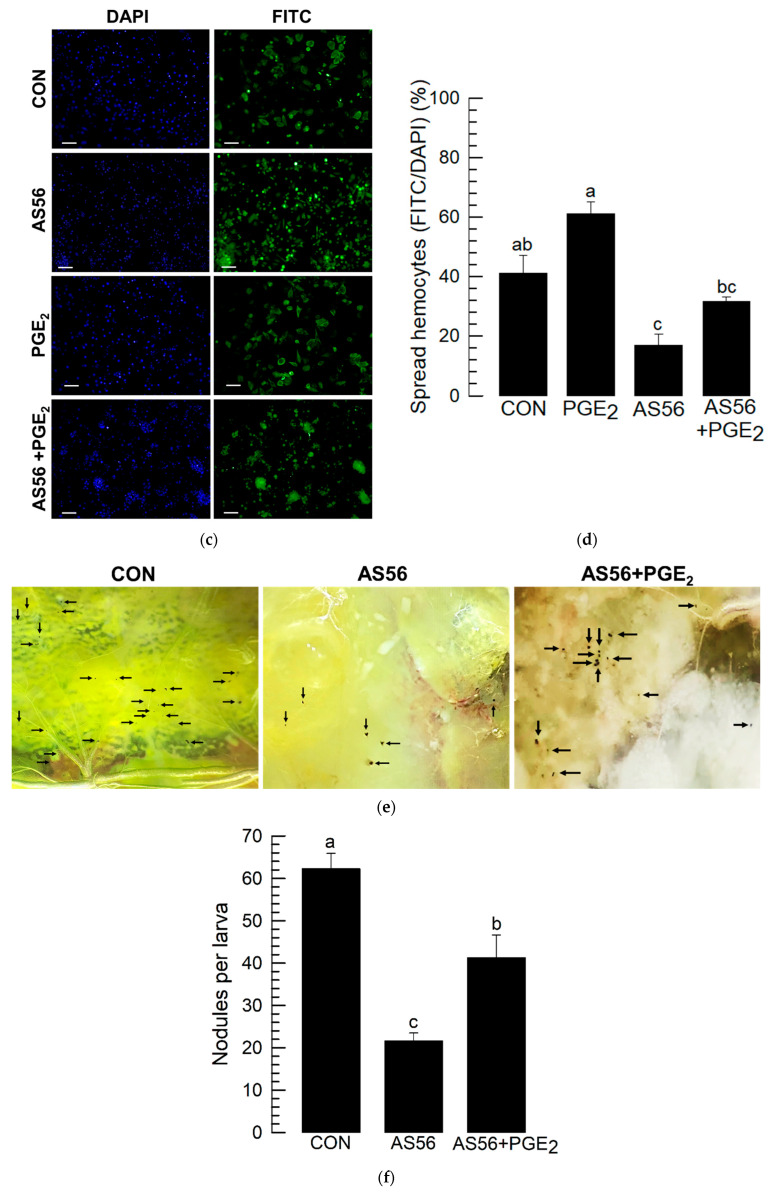
Immunosuppressive activity of AS56 on the cellular immune response of *S. exigua* induced by PGE_2_. (**a**) Inhibitory activity against circulatory hemocyte density measured by total hemocyte count (‘THC’) after AS56 (1 μg/larva) injection. (**b**) Antagonistic activity of AS56 against PGE_2_ on the modulation of THC at 4 h. The control (‘CON’) used a DMSO injection, while AS56 or PGE_2_ was applied at 1 μg/larva. (**c**) Inhibitory activity of AS56 against hemocyte-spreading behavior induced by PGE_2_. Hemocytes were incubated with PGE_2_ (10 µg/mL), AS56 (100 µg/mL), or a mixture of AS56 (100 µg/mL) and PGE_2_ (10 µg/mL) for 20 min. The control (‘CON’) used DMSO. Hemocytes were visualized under a fluorescence microscope at 400× magnification. F-actin filaments were labeled with FITC-conjugated phalloidin (green), and nuclei were counterstained with DAPI (blue). ImageJ software (https://imagej.net/ij/) was used on 24 February 2026 to analyze signal intensities. Scale bar indicates 10 μm. (**d**) Quantification of the spreading behavior by normalizing FITC intensity with DAPI staining. (**e**) Inhibitory effect of AS56 against nodule formation in *S. exigua* in response to a bacterial challenge (5 × 10^4^ *E. coli* cells per larva). Arrows indicate nodules formed in the hemocoel. (**f**) Quantification of nodules. Each treatment was independently replicated three times. Different letters above the standard deviation error bars indicate significant differences among means at Type I error = 0.05 (LSD test).

THC in the hemolymph was estimated to be 7.5 × 10^6^ cells/mL in L5 larvae of *S. exigua* (Figure 1a). When the larvae were injected with AS56, however, they showed a significant (*F* = 42.41; df = 7, 16; *p* < 0.0001) reduction in an incubation-time-dependent manner. In contrast, the addition of PGE_2_ to the larvae increased the THC (Figure 1b). However, the addition of AS56 to the PGE_2_ treatment suppressed the THC down to the uninduced level. This suggested antagonistic activity between these two oxylipins.

Hemocytes spread on non-self surfaces such as a glass plate by extending the F-actin cytoskeleton (Figure 1c). This spreading behavior was significantly stimulated by the addition of PGE_2_. Alternatively, the addition of AS56 suppressed this hemocyte behavior. However, the suppressed hemocyte behavior was rescued by the addition of PGE_2_, supporting their antagonistic activity.

Nodule formation is a cellular immune response against microbial pathogens. In response to a nonpathogenic bacterial infection, the larvae formed around 60 nodules per larva in their hemocoel (Figure 1d). However, the addition of AS56 significantly suppressed this cellular immune response. Again, the addition of PGE_2_ rescued the immunosuppressive larvae.

### 3.2. Comparative Analysis of the Immunosuppressive Activity of AS56 with Its Analogs

**Figure 2:**
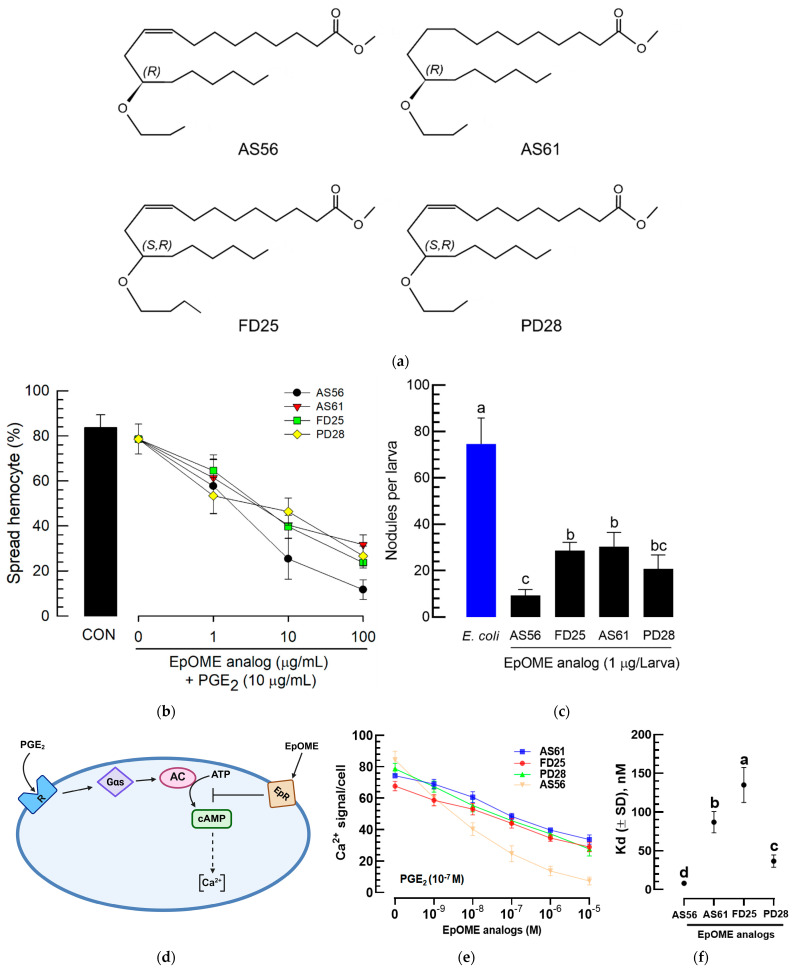
Comparative analysis of immunosuppressive activities of different EpOME analogs in *S. exigua*. (**a**) Chemical structures of four EpOME analogs. (**b**) Antagonistic activities of the EpOME analogs on hemocyte spreading induced by PGE_2_. (**c**) Inhibitory effects of the EpOME analogs on nodule formation in *S. exigua* larvae challenged by *E. coli* (5 × 10^4^ cells per larva, noted by blue bar). (**d**) Antagonistic activities of the EpOME analogs on the upregulation of Ca^2+^ levels in hemocytes induced by PGE_2_. The diagram indicates the modulation of Ca^2+^ levels by two oxylipins through their specific receptors: PGE_2_ receptor (‘R’) and EpOME receptor (‘EpR’). Upon binding of PGE_2_ to its receptor, the small G protein (‘Gαs’) activates adenylate cyclase (‘AC’), which increases the cAMP level. In turn, the upregulated cAMP leads to the upregulation of Ca^2+^. In contrast, EpOME binds to its receptor and inhibits cAMP production, which suppresses the upregulation of Ca^2+^ levels induced by PGE_2_. Hemocytes in the photos were stained with 2 µL of Fura-8 AM (1 mM) for Ca^2+^ detection (green in FITC) and nuclei were stained with DAPI (blue) in the presence of PGE_2_ (10^−7^ M) and EpOME analogs (10^−6^ M). The control (‘CON’) used DMSO treatment. Relative Ca^2+^ signal intensity was quantified as the ratio of Fura-8 to DAPI fluorescence using ImageJ software. (**e**) Suppression of Ca^2+^ levels with increase in EpOME analogs. (**f**) Functional binding affinities of the EpOME analogs to the hemocytes with respect to downregulation of Ca^2+^ levels in the presence of PGE_2_. Each treatment was independently replicated three times. Different letters above the standard error bars indicate significant differences among means at Type I error = 0.05 (LSD test).

To evaluate the changes in the immunosuppressive activity of AS56, three analogs were prepared by deleting the double bond (AS61), by extending the propoxy chain to a butoxy chain (FD25), and by synthesizing a racemic mixture at the alkoxide position (PD28) (Figure 2a). Using these four synthetic analogs, their inhibition of hemocyte-spreading behavior was assessed (Figure 2b). At 10 µg/mL, hemocytes were activated by the addition of PGE_2_ and additionally treated with an EpOME analog. All four analogs significantly (*F* = 16.59; df = 3, 26; *p* < 0.0001) suppressed the hemocyte behavior. However, these four analogs differed in their suppressive activities against hemocyte behavior. Although they were not different at a low concentration (1 µg/mL), they were significantly different at higher doses, in which AS56 was the most potent compared to the other three analogs (*F* = 42.62; df = 4, 10; *p* < 0.0001). These four analogs were also compared in their ability to suppress hemocytic nodule formation, in which AS56 was the most potent compared to the other three analogs (Figure 2c).

To clarify the inhibitory activity of the EpOME analogs at the cellular level, Ca^2+^ levels were monitored in hemocytes stimulated by PGE_2_ (Figure 2d). The addition of the EpOME analogs reduced the Ca^2+^ signal (Figure 2e). When the functional binding affinities of the EpOME analogs to the hemocyte preparation were estimated, AS56 exhibited the lowest K_D_ value (8.0 ± 1.2 nM) among the analogs (Figure 2f).

### 3.3. Comparative Analysis of the Cytotoxicity of AS56 with Its Analogs

**Figure 3:**
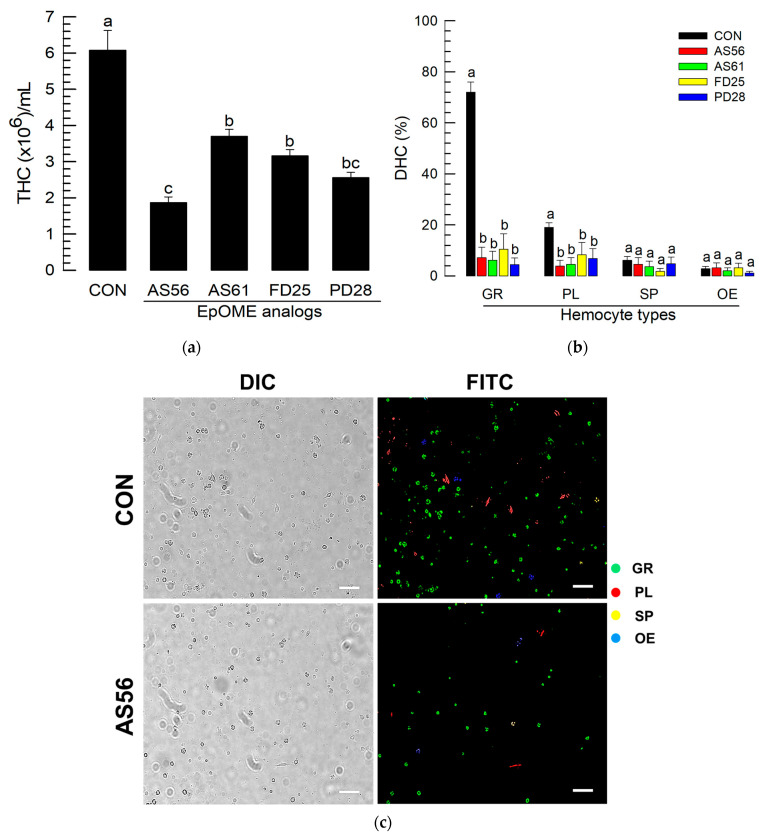
Comparative analysis of hemocyte toxicities of EpOME analogs in *S. exigua*. (**a**) Suppression of total hemocyte count (‘THC’) at 4 h post-injection of analogs (1 μg/larva). (**b**) Changes in differential hemocyte counts (‘DHC’) following treatments: granulocyte (‘GR’), plasmatocyte (‘PL’), spherulocyte (‘SP’), and oenocytoid (‘OE’). Each treatment was independently replicated three times. Different letters above the standard error bars indicate significant differences among means at Type I error = 0.05 (LSD test). (**c**) Identification of hemocyte types using fluorescence in situ hybridization (FISH) with cell-type-specific probes. The FISH probes targeting specific marker genes include *cecropin B1* for GR (green), *paired mesoderm homeobox protein 2A-like* for PL (red), *prophenoloxidase-2* for OE (blue), and *Repat9* for SP (yellow). Scale bar represents 10 μm.

When the EpOME analogs were injected into larvae, all four analogs reduced total hemocyte counts, with AS56 being the most potent (Figure 3a). When the hemocytes were classified into four morphological types to estimate DHC, the cytotoxic effects were biased toward GR and PL in the AS56 treatment (Figure 3b). This was further supported by cell-type-specific FISH probe analysis, in which the two cell types were markedly reduced (Figure 3c).

**Figure 4:**
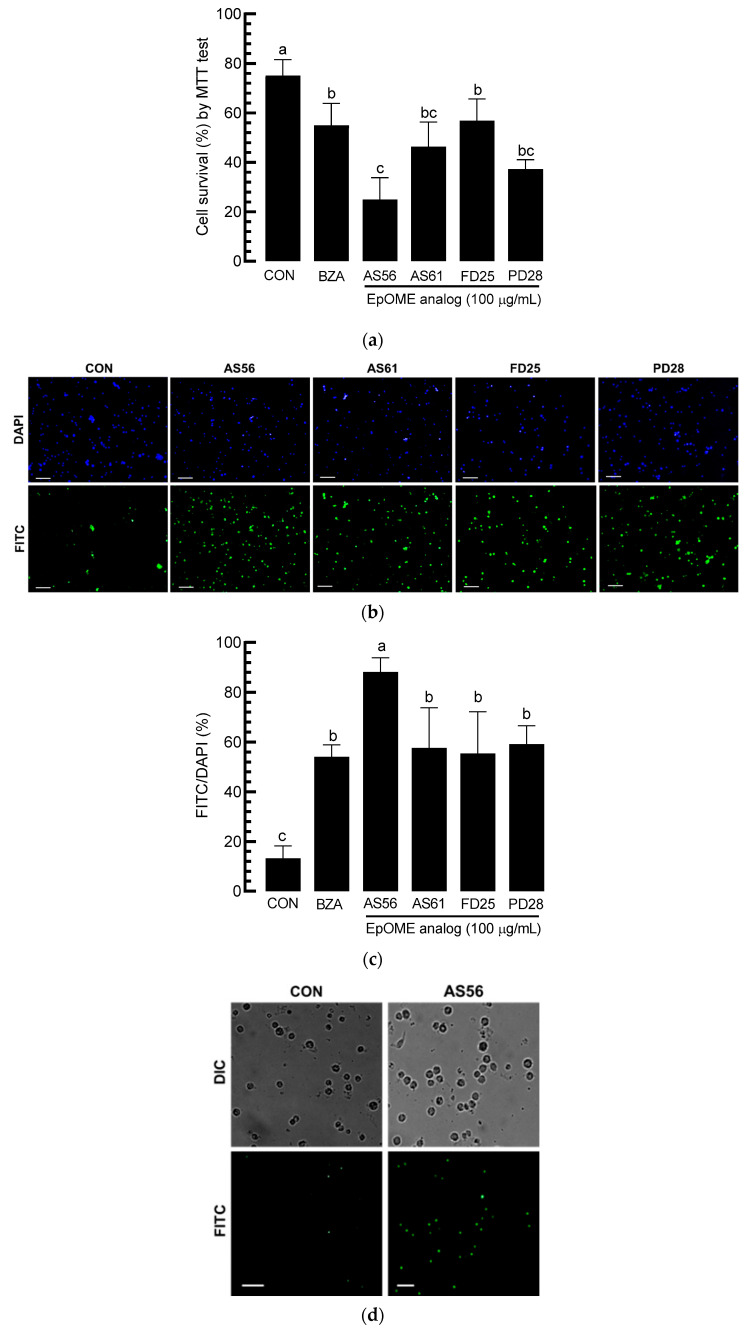
Comparative cytotoxicity of EpOME analogs against insect cells. (**a**) Cell viability assessed by the MTT assay. Sf9 cells were treated with EpOME analogs (100 µg/mL) and benzylideneacetone (‘BZA’) (1 µM) as a positive control for 24 h at 28 °C. Absorbance was measured at 570 nm after dissolution of formazan crystals in DMSO. Cell viability is expressed as a percentage relative to untreated control cells (‘CON’). (**b**) Induction of apoptosis in hemocytes by the analogs. Apoptotic cells were detected by BrdU incorporation using the TUNEL assay. Representative fluorescence microscopy images show nuclei stained with DAPI (blue) and apoptotic cells labeled with FITC-conjugated BrdU (green). *S. exigua* hemocytes were treated with EpOME analogs (100 µg/mL) and BZA (1 µM) as a positive control. Scale bar represents 10 μm. FITC-positive cells were quantified by normalization with total DAPI-stained nuclei. (**c**) Quantification of apoptosis by normalizing FITC intensity with DAPI staining. Each treatment was independently replicated three times. Different letters above standard error bars indicate significant differences among means at Type I error = 0.05 (LSD test). (**d**) Effect of AS56 (100 µg/mL) in inducing apoptosis in hemocytes assessed by an immunofluorescence assay using cleaved caspase-3 (Asp175)-specific antibodies. The cells were visualized one hour after treatment and apoptotic signals were detected using FITC under the fluorescence microscope. Each replication used individual adults and independently replicated three times. Different letters above the standard error bars indicate significant differences among means at Type I error = 0.05 (LSD test). Scale bar = 10 μm.

Cytotoxicity was evaluated by the MTT test to assess the viability of the hemocytes after incubation with the EpOME analogs (Figure 4a). As a positive control, BZA was used for the cytotoxicity test because it showed a potent cytotoxicity by inducing apoptosis [27]. Here, AS56 was the most potent in terms of cytotoxicity among the analogs. In the TUNEL assay, AS56 treatment highly induced the DNA fragmentation and was the most potent with regard to apoptosis induction among the analogs (Figure 4b). To support the apoptotic cytotoxicity of AS56, the IFA specific to cleaved Capase-3 was performed and showed a marked apoptosis activity against hemocytes of *S. exigua* (Figure 4c).

### 3.4. Comparative Analysis of the Insecticidal Activity of AS56 with Its Analogs

**Figure 5:**
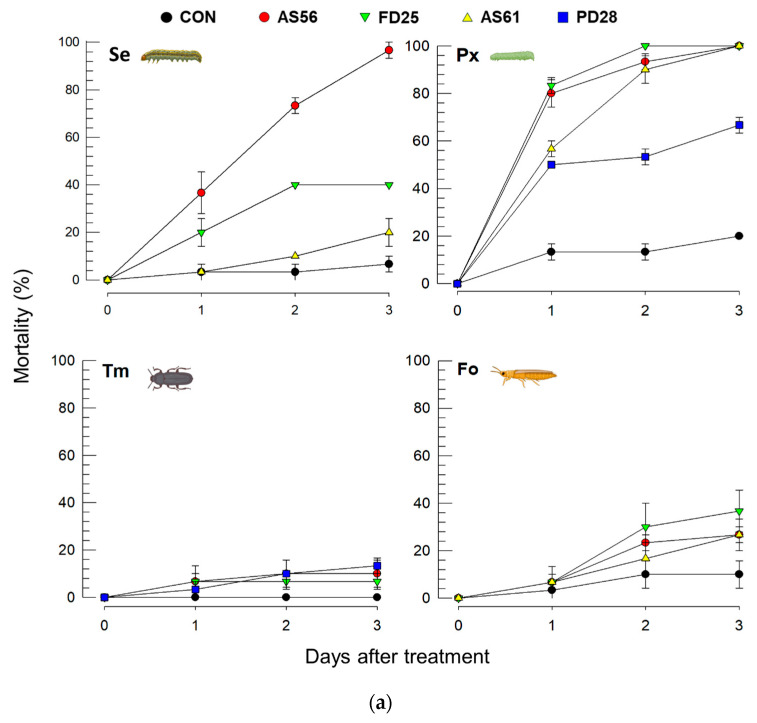

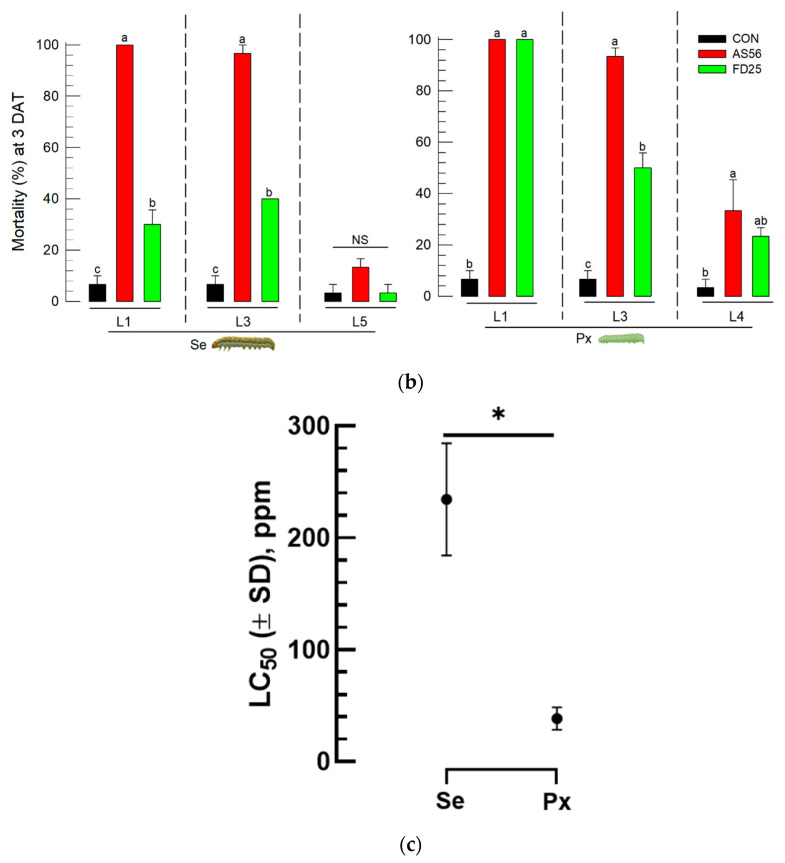
Insecticidal bioassays of EpOME analogs using a leaf-dipping method. (**a**) Specificity of the EpOME analogs (1000 ppm) in the insecticidal activities. Test insects include two lepidopterans (*S. exigua* (‘Se’) and *P. xylostella* (‘Px’)), one coleopteran (*T. molitor* (‘Tm’)), and one thysanopteran (*F. occidentalis* (‘Fo’)). All test insects were tested at the L2 stage, except for the young (<24 h after emergence) adult stage for Fo. Each experimental unit was a Petri dish containing 10 test insects and was replicated three times. The control (‘CON’) represents a leaf dipping in water. ‘NS’ = non-significant. (**b**) Comparison of two analogs (AS56 and FD25, 1000 ppm) in the insecticidal activities against two lepidopteran species at different larval stages (‘L1–L5’). Mortality was calculated at 3 days after treatment (‘DAT’). Different letters above standard error bars indicate significant differences among means at Type I error = 0.05 (LSD test). (**c**) Median lethal concentrations (LC_50_ values) of AS56 against two lepidopteran insects at the L2 stage. Each treatment was independently repeated three times. Asterisks above the standard deviation (‘SD’) bars indicate significant differences between means at Type I error = 0.05 (LSD test).

The four EpOME analogs were potent against lepidopteran insects (*S. exigua* and *P. xylostella*) but not against coleopteran (*T. molitor*) or thrips (*F. occidentalis*) species in a leaf-dipping bioassay (Figure 5a). Among the EpOME analogs, AS56 was the most toxic to *S. exigua*. In *P. xylostella* larvae, FD25 was also highly toxic. To clarify their relative toxicities, these two analogs were applied to different larval stages (Figure 5b). In *S. exigua*, AS56 was more toxic to L1 and L3 than FD25. However, both analogs did not show significant toxicity to L5 larvae. In *P. xylostella*, AS56 was more toxic than FD25 in L3, but showed no difference in L1 and L5. These assays indicate that AS56 is the most potent in insecticidal activity against the lepidopteran species. AS56 showed a typical dose-dependent insecticidal activity against both lepidopteran insects (Figure S1). It was more potent against *P. xylostella* than *S. exigua* with regard to LC_50_ (Figure 5c). LC_50_ values at 3 days post-treatment were 231.2 ppm (95% CI: 148.7–322.8 ppm, χ^2^ = 27.8, R^2^ = 0.8184) for *S. exigua* and 37.6 ppm (95% CI: 21.8–56.3 ppm, χ^2^ = 40.69, R^2^ = 0.6557) for *P. xylostella*.

### 3.5. Behavioral Alteration of AS56-Intoxicated Larvae

**Figure 6:**
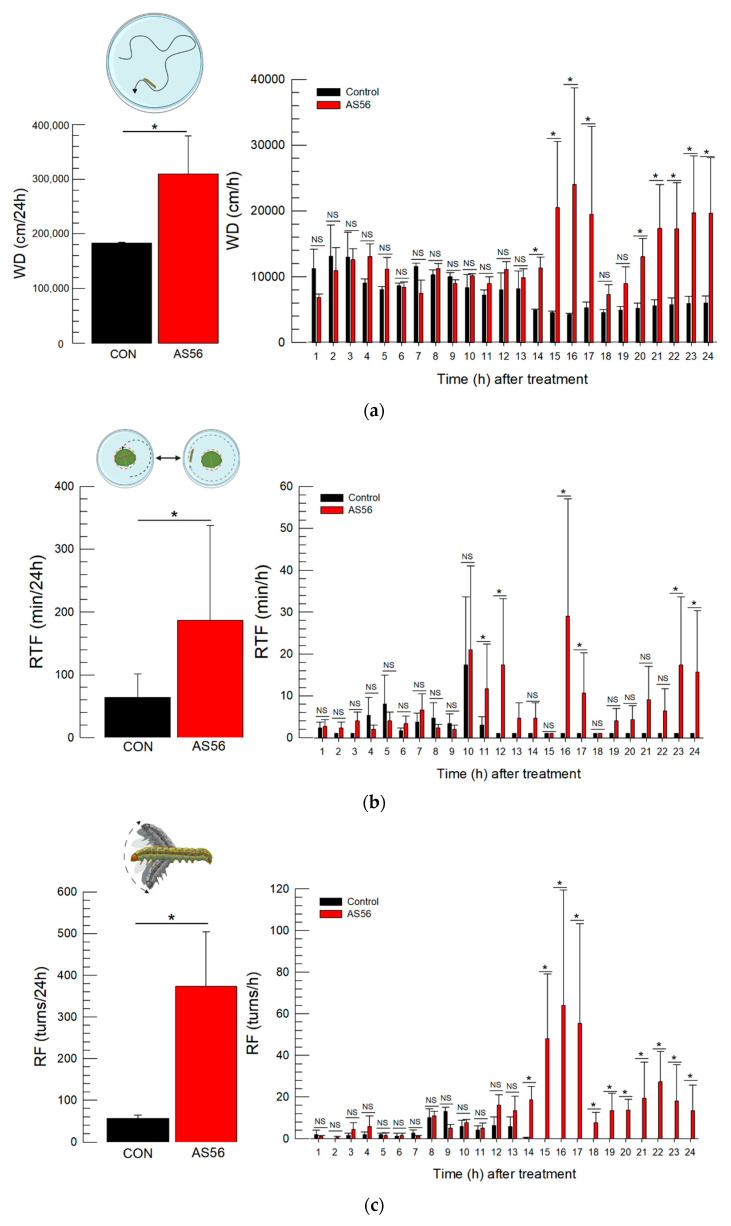
Behavioral analysis of AS56-intoxicated larvae of *S. exigua*. The three different behavioral patterns assessed are (**a**) walking distance (‘WD’), (**b**) retention time in the food zone (‘RTF’), and (**c**) rotation frequency (‘RF’). Each behavior was assessed hourly over 24 h. Each treatment was independently repeated three times. Asterisks above the standard error bars indicate significant differences among means at Type I error = 0.05 (LSD test). ‘NS’ = Non-significant.

To assess the acute toxic activity of AS56, larval behavior was continuously monitored for 24 h and analyzed for specific parameters (Figure 6). The distance of larval locomotion was assessed every hour (Figure 6a). Untreated larvae exhibited continuous movement at an average of 10,000 cm/h. The AS56-treated larvae also showed a similar speed of movement until 13 h. After that, the treated larvae exhibited significantly higher locomotion speeds, resulting in about 1.5 times longer movement distance than the control larvae.

To measure dispersion from the diet, because our previous study showed that AS56 treatment reduced the feeding amount of the larvae [24], returning behavior to the diet was counted (Figure 6b). AS56-treated larvae frequently moved away from the diet, while control larvae stayed near the diet. Thus, the frequency of returning to the diet was threefold greater in the AS56-treated larvae than in the control larvae. The behavioral alteration was observed as early as 11 h after the treatment.

Searching behavior represents the shaking of the larval head, which contains the antennae. This behavior also increased about six-fold after AS56 intoxication (Figure 6c). The increase was observed at 14 h post-treatment with AS56.

### 3.6. Control Efficacy of AS56 in Cabbage Fields

**Figure 7:**
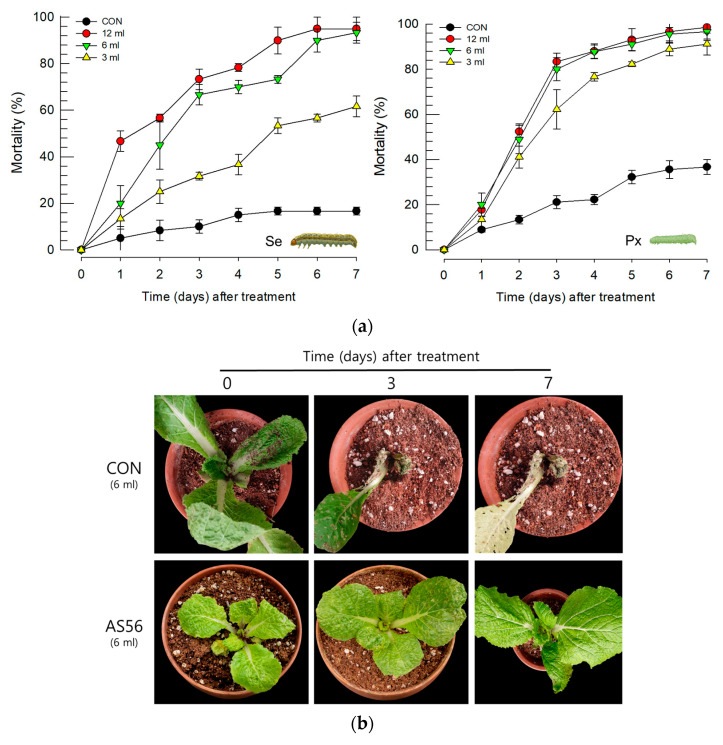

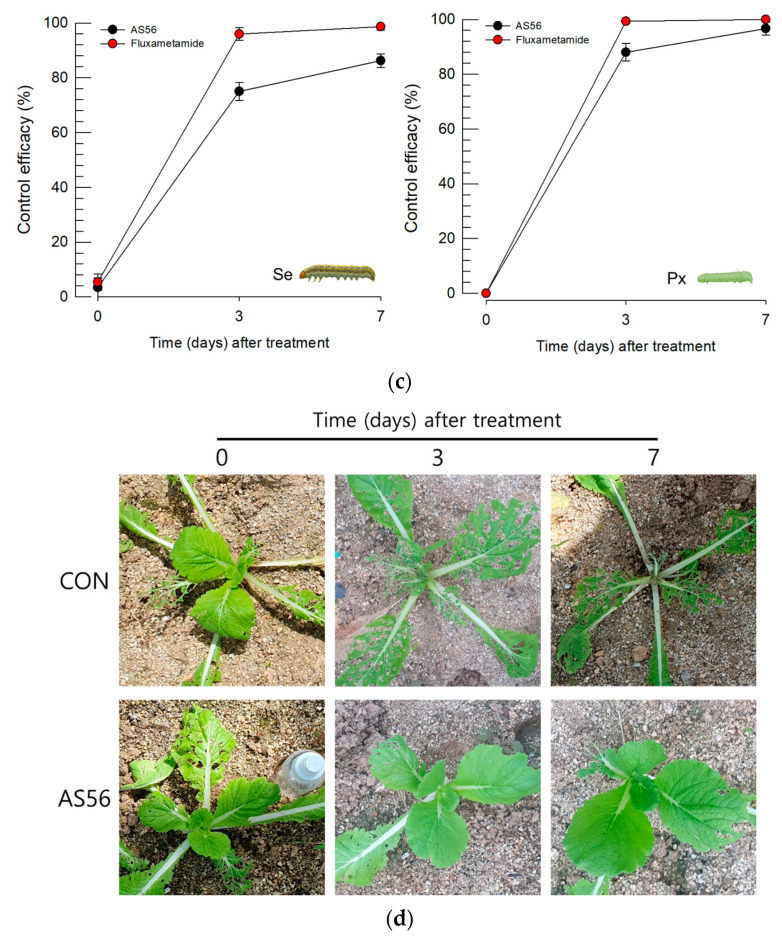
Application of AS56 at 500 ppm against cabbage fields infested with *S. exigua* (‘Se’) or *P. xylostella* (‘Px’). The control (‘CON’) represents a water spray without any test compound. (**a**) A pot assay was conducted to determine the spraying amount. Each cabbage was sprayed with three different volumes. Each pot contained a cabbage infested with 30 young (L2–L3) larvae. (**b**) Representative symptoms after treatment with the pot assay. (**c**) A field assay was conducted to test the control efficacy of AS56. A commercial insecticide, fluxametamide (500 ppm), was sprayed at the same volume (6 mL per plant). Control efficacies were estimated based on surviving insects by comparing them with the survival numbers in the control. Experimental units were allocated in a randomized block design. (**d**) Representative symptoms after treatment with the field assay. Each treatment was replicated three times.

The leaf-dipping method determined the effective lethal concentrations of AS56 to be 500 ppm against *S. exigua* and 500 ppm against *P. xylostella*. Using these lethal concentrations, we tested the spraying volume required per cabbage plant through pot assays (Figure 7). The insecticidal activity increased with an increase in the spraying volume. For both insects, 6 mL per plant was ideal because there was no significant difference in control efficacy compared to the 12 mL spray. Using this 6 mL spraying volume at 500 ppm, AS56 was applied to cabbage plants infested by *S. exigua* (Figure 7a). A commercial insecticide, fluxametamide, yielded over 95% control efficacy at 3 days after treatment and maintained this control efficacy after 7 days. AS56 also showed high control efficacy (~75%) at 3 days after treatment, with a slight increase (~85%) at 7 days after treatment. In *P. xylostella* (Figure 7b), fluxametamide also showed almost 100% control efficacy. AS56 at 500 ppm showed almost 88% control efficacy at 3 days after treatment and increased its control efficacy to over 95% at 7 days after treatment. Two-way ANOVA revealed significant differences between AS56 and fluxametamide treatments in both field experiments (Table S2).

## 4. Discussion

EpOMEs play a crucial role in suppressing the insect immune response at the late infection stage to return the system to a normal condition, because excessive and unnecessary immune responses are usually detrimental [8]. The induced cAMP signal by PGE_2_ was reduced by EpOME probably inhibiting adenylate cyclase via an independent EpOME receptor (unpublished data). To suppress cellular immune responses, EpOME usually induces apoptosis of hemocytes to reduce excessive hemocyte numbers in the circulatory system [24]. This cytotoxic activity of EpOMEs provides a basis for developing a novel insecticide. Thus, the addition of any exogenous EpOME analog may give rise to fatal cytotoxicity in target insects. This hypothesis was tested in this study using different EpOME analogs.

The chemical structure of AS56 is critical to its action as a potent EpOME analog. AS56 was selected as a potent EpOME analog by screening different alkoxide derivatives such as methoxy, ethoxy, propoxy, and butoxy alkoxides [24]. Also, the *R*-type stereoisomer at the twelfth carbon was more potent than the *S*-type isomer in immunosuppression. In our current study, AS56 antagonized the immune-mediating effect of PGE_2_. This antagonistic activity of AS56 was used to compare chemically modified analogs of AS56: AS61, FD25, and PD28. AS61 lacks the double bond between carbon atoms 9 and 10. FD25 is a 1:1 mixture of both enantiomers of AS56, and PD28 is similar to FD25, but the propoxy group is replaced with a butoxy group. AS61 lost antagonistic activity against PGE_2_ compared to AS56 with respect to two cellular immune responses, namely hemocyte-spreading behavior and nodulation. Furthermore, the functional binding affinity of AS61 to hemocytes was significantly reduced compared to AS56 by almost 18-fold. The receptor to EpOME is identified and expressed in several tissues, including hemocytes of *S. exigua* (unpublished data). This suggests the critical role of the double bond, probably due to the three-dimensional configuration of the molecule forming a kink structure caused by the cis configuration of the double bond, which allows for a stable and strong interaction with its membrane EpOME receptor. Both FD25 and PD28 also exhibited low antagonistic activity against PGE_2_ compared to AS56. One of the reasons may be the mixture of stereoisomers, including the less potent *S*-enantiomer. PD28 was superior to FD25, supporting our previous study [24], indicating that the propoxy alkoxide (=PD28) was better than the butoxy alkoxide (=FD25) in biological activity. These results indicate that the double bond between carbon atoms 9 and 10, as well as the *R*-configuration of the stereogenic center at twelfth position, are crucial components for AS56 to exert its inhibitory activity against insect immune responses by antagonizing PGE_2_ modulation. The critical significance of these two chemical components of AS56 was also supported by its cytotoxicity. That is, any change in these two components significantly impaired cytotoxicity against hemocytes. In particular, granulocytes were highly susceptible to the cytotoxicity of AS56. It is interesting that granulocytes highly expressed the PGE_2_ receptor gene, as shown by single-cell RNA-Seq of *S. exigua* hemocytes [15], explaining the cell-specific toxicity of AS56. However, the crosstalk between PGE_2_ and EpOMEs in the target cells needs to be further analyzed with respect to the induction of apoptosis.

AS56 exhibits specific insecticidal activity against lepidopteran species. AS56 is an analog of EpOME. In mammals, EpOMEs have been known to act as leukotoxins because they cause an increase in leukocyte cytotoxicity in response to bacterial infections [7]. However, Moghaddam et al. [28] demonstrated that EpOME cytotoxicity was, in fact, a result of sEH metabolism into DiHOMEs. Although intravenous injection of leukotoxin in healthy mice caused acute respiratory distress syndrome (ARDS), inhibition of sEH prevented this toxicity. Indeed, direct administration of DiHOMEs resulted in the same ARDS phenotype [29]. However, DiHOMEs are inactive metabolites of their corresponding EpOMEs, because DiHOMEs are no longer as active in immunosuppressive and cytotoxic activities as EpOMEs in *S. exigua* [9]. This suggests differential toxicity of AS56 between insects and mammals. Its insecticidal activity may be derived from its immunosuppressive activity by antagonizing PGE_2_ mediation in insects.

Immune suppression alone cannot explain AS56-induced mortality, as bioassays were performed without pathogen infection. Thus, additional mechanisms, such as metabolic disruption via PGE_2_ antagonism and direct cytotoxicity on gut epithelium, likely contributed to the insecticidal activity. It should be noted that the relationship between immunosuppression and insect mortality observed here is correlative, and further studies are needed to establish causality. Alternatively, its toxicity may be explained by its antagonistic activity against PGE_2_, which mediates several physiological processes other than immunity in insects [2]. PGs act as downstream signals of hypertrehalosemic hormones, HTH-1 and HTH-2, in the cockroach, *Periplaneta americana*, because a COX inhibitor, used to inhibit PG biosynthesis, suppresses the release of trehalose from the fat body, which is activated by HTH treatment [30]. Indeed, PG treatment led to dose-dependent increases in trehalose efflux from trophocytes [31]. The role of PGE_2_ in metabolism was further supported by a mutant lacking the PGE_2_ receptor in *S. exigua*, which suffered from a marked retardation in larval development [4]. Thus, the insecticidal activity of AS56 can be explained by its interference with the metabolism required for insect development.

AS56 exhibited acute toxicity in *S. exigua* by altering larval behavior associated with feeding. These behavioral aberrations included hyper-locomotor activity and were observed around 12 h post-injection of AS56. These behavioral changes were followed by the insecticidal activity of AS56, because AS56 induced significant mortality as early as 24 h after oral treatment. This observation suggests that AS56 enhances neural activity to induce unnecessary movements, leading to dysregulated locomotion. The wide distribution of the EpOME receptors among the different tissues (unpublished data) suggests the induced behavior by AS56 directly on the nervous system or neuromuscular junction to alter the behavior. Furthermore, our previous loss-of-function approach [4,9] in EpOME/PGE_2_ signal induced significant dysregulation of immature development and reproduction of *S. exigua*. In mammals, oxylipins, including eicosanoids and EpOMEs, play crucial roles in mediating pain by activating G-protein coupled receptors (GPCRs) and/or modulating the activity of ion channels, including the transient receptor potential cation channel subfamily vanilloid 1 (TRPV1) channel, which leads to increased activity of peripheral sensory neurons [32,33]. TRPs play an important role in various sensing mechanisms, including vision, taste, smell, hygrosensation, and thermosensation [34]. Five TRPs in *D. melanogaster* are known to be associated with temperature sensing; painless, pyrexia, and trpA1 are involved in high-temperature sensing, and trp and trpL in low-temperature sensing [35,36,37,38]. These findings suggest that AS56 may activate TRPs and enhance unnecessary locomotor activity in *S. exigua*. However, the casual link between EpOME and TRP in *S. exigua* needs to be clarified in future study. Altogether, 12,13-EpOME was modified into a potent analog by stereo-specific propoxy replacement. This analog exhibit oral toxicity and is specifically insecticidal against lepidopteran insects with an oral toxicity. To be applied for development of a novel insecticides, it needs subsequent analyses, such as metabolic fates in insects, phytotoxicity, and other toxicological assays against mammals and beneficial/nontarget organisms. In addition, the residual efficacy test, along with other commercial insecticides, needs to be added.

## Data Availability

The original contributions presented in the study are included in the article/Supplementary Material; further inquiries can be directed to the corresponding author.

## Supplementary Materials

The following supporting information can be downloaded at: https://www.mdpi.com/article/10.3390/insects17060588/s1. Figure S1: Bioassays of AS56 using a leaf-dipping method against L2 larvae of *S. exigua* (‘Se’) and *P. xylostella* (‘Px’). Each experimental unit was a Petri dish containing 10 test insects and was replicated three times. The control (‘CON’) represents leaf dipping in water. Mortality was calculated at 3 days after treatment (‘DAT’). Table S1: FISH probes for hemocyte identification: granulocytes (‘GR’), plasmatocytes (‘PL’), spherulocytes (‘SP’), and oenocytoids (‘OE’). Specific marker genes include *cecropin B1* (‘CecB1’), *paired mesoderm homeobox protein 2A-like* (‘PMH’), *prophenoloxidase-2* (‘PPO2’), and *Repat9* (‘REPAT9’). Table S2: Two-way ANOVA results for control efficacy of AS56 and fluxametamide against *Spodoptera exigua* and *Plutella xylostella* in pot and field experiments.
