# Supplementary material for: Chemical Modifications of an Insect Immune Resolvin, EpOME, to a Broad-Spectrum Lepidopteran-Specific Insecticide
Source: Insects. 2026 Jun 4;17(6):588. doi: 10.3390/insects17060588 (PMC13300193; doi:10.3390/insects17060588)
Supplement: Supplementary file 1 [file insects-17-00588-s001.zip › insects-4263805-supplementary.pdf]

Supplementary Information

Table S1. FISH probes for hemocyte identification: granulocytes ('GR'), plasmatocytes ('PL'), spherulocytes ('SP'), and oenocytoids ('OE'). Specific marker genes include *cecropin B1* ('CecB1'), *paired mesoderm homeobox protein 2A-like* ('PMH'), *prophenoloxidase-2* ('PPO2'), and *Repat9* ('REPAT9').

Table S2. Two-way ANOVA results for control efficacy of AS56 and fluxametamide against *Spodoptera exigua* and *Plutella xylostella* in pot and field experiments.

Figure S1. Bioassays of AS56 using a leaf-dipping method against L2 larvae of *S. exigua* ('Se') and *P. xylostella* ('Px'). Each experimental unit was a Petri dish containing 10 test insects and was replicated three times. The control ('CON') represents leaf-dipping in water. Mortality was calculated at 3 days after treatment ('DAT').

Table S1. FISH probes for hemocyte identification: granulocyte ('GR'), plasmatocyte ('PL'), spherulocyte ('SP'), and oenocytoid ('OE'). Specific marker genes include *cecropin B1* (CecB1), *paired mesoderm homeobox protein 2A-like* (PMH), *prophenoloxidase-2* (PPO2), and *Repat9* (REPAT9).

Genes	Direction	Sequence (5'-3')	Uses
**CecB1**	Antisense	FAM-TTTTCCCGAGTGTTTCTGTTCGT	FISH for GR
	Sense	FAM-AAAACGAACAGAAACACTCGGGA	
**PMH**	Antisense	FAM-TTTTTGGCGACCTTTACCAGCAT	FISH for PL
	Sense	FAM-AAAATGCTGGTAAAGGTCGCCAA	
**PPO2**	Antisense	FAM-TTTCACGGTCGGAGAAGTCCAAA	FISH for OE
	Sense	FAM-AAATTTGGACTTCTCCGACCGTG	
**REPAT9**	Antisense	FAM-TTTGCAAACCTTCACCGAGTCGT	FISH for SP
	Sense	FAM-AAAACGACTCGGTGAAGGTTTGC	

Table S2. Two-way ANOVA results for control efficacy of AS56 and fluxametamide against *Spodoptera exigua* and *Plutella xylostella* in pot and field experiments.

Pot experiment					
S. exigua					
**Source**	**df**	**SS**	**MS**	**F value**	**P value**
**TRT**	3	6.8118	2.2706	145.35	<.0001
**DATE**	7	9.8759	1.4108	90.31	<.0001
**TRT*DATE**	21	1.5312	0.0729	4.67	<.0001
**Error**	64	0.9998	0.0156		
**Total**	95	19.2186			
P. xylostella					
**Source**	**df**	**SS**	**MS**	**F value**	**P value**
**TRT**	3	3.5498	1.1833	135.84	<.0001
**DATE**	7	12.0397	1.7200	197.45	<.0001
**TRT*DATE**	21	1.13689	0.0541	6.21	<.0001
**Error**	64	0.5575	0.0087		
**Total**	95	17.2838			

Field experiment					
S. exigua					
**Source**	**df**	**SS**	**MS**	**F value**	**P value**
**TRT**	1	0.2805	0.2805	15.49	0.0020
**DATE**	2	5.2206	2.6104	144.17	<.0001
**TRT*DATE**	2	0.0645	0.0323	1.78	0.2101
**Error**	12	0.2173	0.0181		
**Total**	17	5.7828			
P. xylostella					
**Source**	**df**	**SS**	**MS**	**F value**	**P value**
**TRT**	1	0.0984	0.0984	17.06	0.0014
**DATE**	2	8.2991	4.1495	719.54	<.0001
**TRT*DATE**	2	0.0679	0.0339	5.88	0.0166
**Error**	12	0.0692	0.0058		
**Total**	17	8.5345			

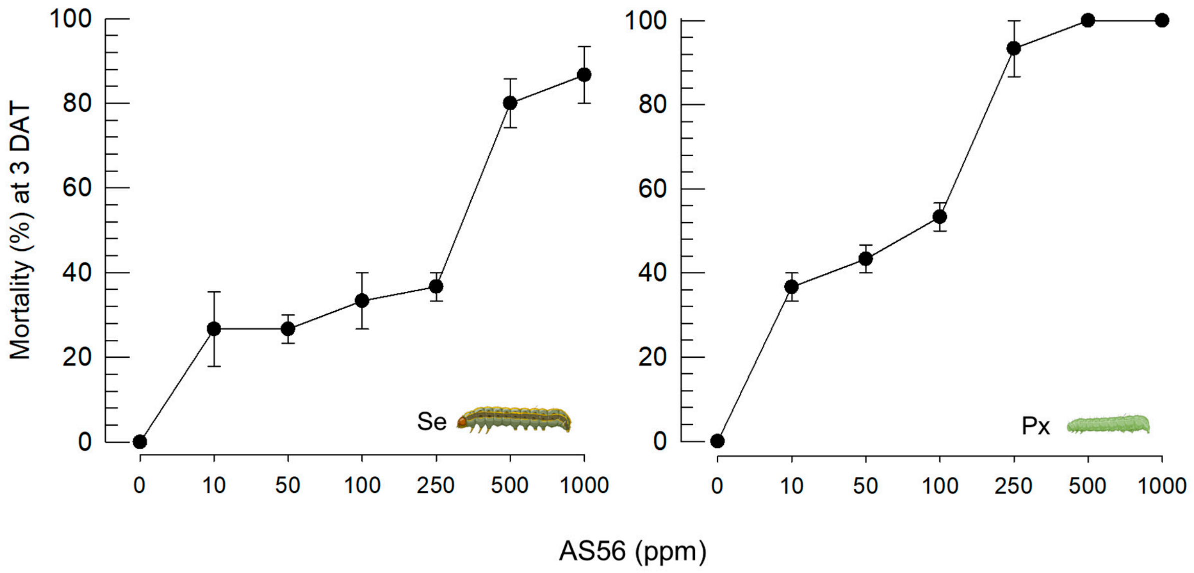
100
80
60
40
20
0
Mortality (%) at 3 DAT
Se
0
10
50
100
250
500
1000
100
80
60
40
20
0
Px
0
10
50
100
250
500
1000
AS56 (ppm)

Figure S1.

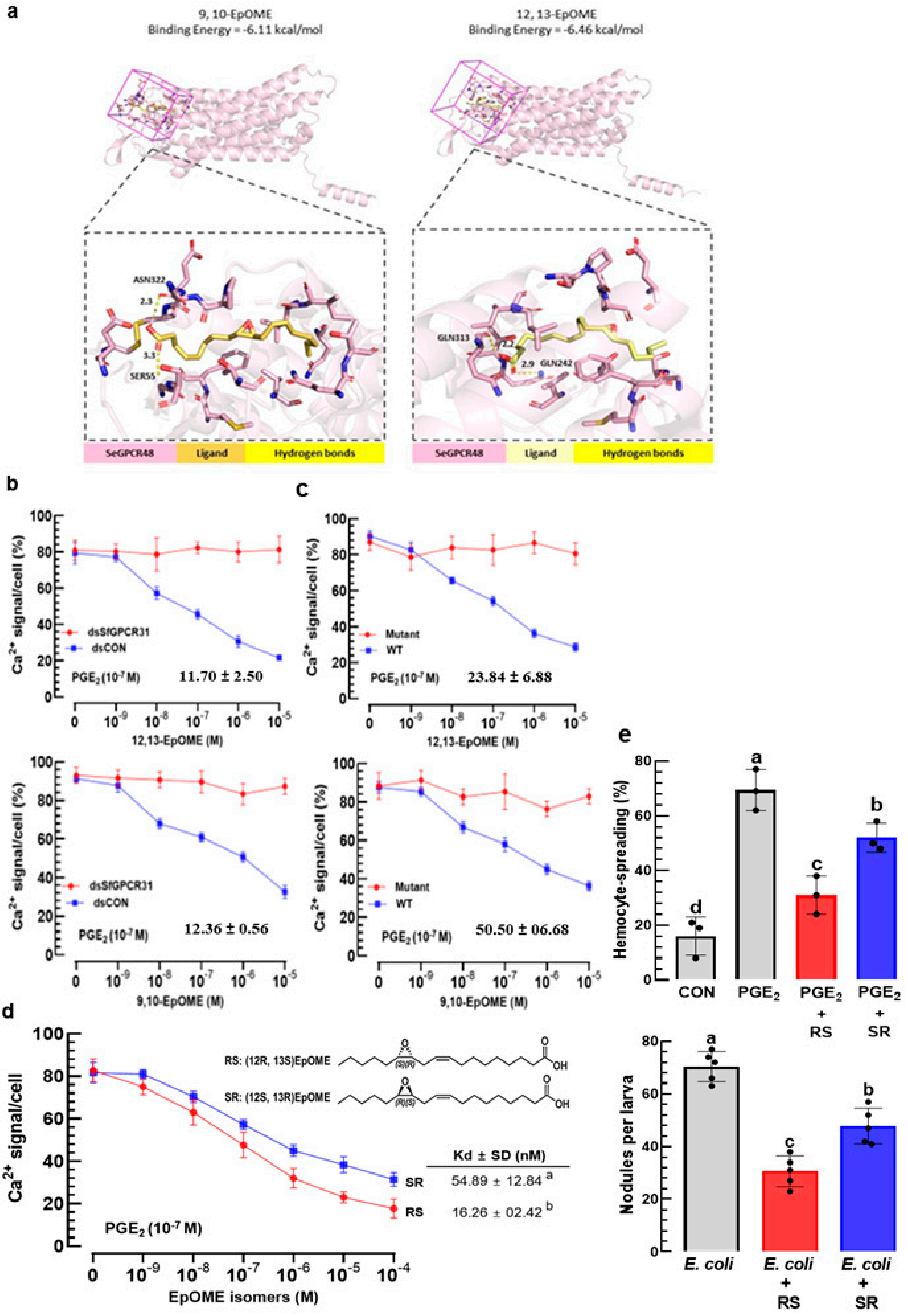
a
9, 10-EpOME
Binding Energy = -6.11 kcal/mol
12, 13-EpOME
Binding Energy = -6.46 kcal/mol
ASN322
SER55
GLN313
GLN242
SeGPCR48
Ligand
Hydrogen bonds
b
Ca2+ signal/cell (%)
dsSfGPCR31
dsCON
PGE2 (10-7 M)
11.70 ± 2.50
12,13-EpOME (M)
12.36 ± 0.56
9,10-EpOME (M)
c
Mutant
WT
23.84 ± 6.88
50.50 ± 06.68
d
Ca2+ signal/cell
RS: (12R, 13S)EpOME
SR: (12S, 13R)EpOME
Kd ± SD (nM)
SR 54.89 ± 12.84 a
RS 16.26 ± 02.42 b
PGE2 (10-7 M)
EpOME isomers (M)
e
Hemocyte-spreading (%)
CON
PGE2
PGE2 + RS
PGE2 + SR
Nodules per larva
E. coli
E. coli + RS
E. coli + SR
